# Osteochondral Injury of the Talus Treated With Cell-Free Hyaluronic Acid-Based Scaffold (Hyalofast®) – A Reliable Solution

**DOI:** 10.7759/cureus.17928

**Published:** 2021-09-13

**Authors:** Mohd Yazid Bajuri, Suffian Sabri, Norliyana Mazli, Faris Aiman Sarifulnizam, Husna Mohd Apandi

**Affiliations:** 1 Orthopaedics and Traumatology, Universiti Kebangsaan Malaysia Medical Centre, Kuala Lumpur, MYS

**Keywords:** cell-free hyaluronic acid-based scaffold, ankle injuries, osteochondral lesions, microfracture, open medial malleolus osteotomy

## Abstract

Background: Osteochondral injuries commonly occur in load-bearing joints, mainly caused by traumatic incidents that can lead to detachment of the cartilage fragment either partial or complete.

Objective: This study aims to review the long-term outcome of osteochondral injury of the talus treated with a cell-free hyaluronic acid-based scaffold (Hyalofast®, Anika Therapeutics Inc., Bedford, Massachusetts, USA).

Method: This study evaluated the data of seven patients who underwent medial malleolus osteotomy, microfracture, and cell-free hyaluronic acid-based scaffold (HYALOFAST®) insertion between 2015 to 2018. All patients had an osteochondral lesion (OCL) grade III and IV of the talus based on Dipaola classification due to trauma. They were followed up for at least two years and assessed by the short form 36 health survey questionnaire (SF36) for both physical functioning and mental health, American Orthopaedic Foot and Ankle Society (AOFAS) scoring system, and visual analog scale (VAS).

Result: All patients were satisfied in terms of physical function, mental health, and pain after one month of surgery (p-value<0.05). There was also an improvement in AOFAS hindfoot and VAS scores from preoperative to postoperative. No complications were noted in the surgical site or bone union.

Conclusion: Medial malleolus osteotomy, cell-free hyaluronic acid-based scaffold (HYALOFAST®) grafting, and microfracture are considered relatively easy techniques that are a good choice for patients with sizeable cartilage deficiency and provide a good functional outcome.

## Introduction

Osteochondral injuries are regularly seen in the load-bearing joints. An osteochondral ankle defect is the abnormal articular cartilage of the talus and damage to the bone surrounding it which is mainly caused by traumatic incidents such as sports injury and motor vehicle accidents. These traumatic occurrences can lead to detachment of the cartilage fragment that can be either partial or complete [1,2].

The diagnosis of acute ankle osteochondral injuries is usually missed because the injury is not apparent and causes minimal or no functional limitation. It is one of the frequent causes of ankle nuisance and is commonly encountered in young sportspersons after ankle injury [1,2]. Other reasons being speculated that might lead to this problem include genetic factors, osteonecrosis, and endocrine disease whose etiology cannot be explained [3]. Deficiencies at the cartilage region will produce deep ankle pain during load-bearing activities. A reduction in the function of the ankle and range of movement, catching sensation, worsening stiffness, swelling, and locking may be present. The diagnosis is usually based on the history, clinical findings, and imaging investigation by X-ray and magnetic resonance imaging (MRI) [4].

The standard treatment strategies of symptomatic osteochondral lesions can be divided into conservative treatment and surgical interventions. Conservative options include rest and elevating the affected limb, application of a cast, and analgesic. Surgical intervention options for these injuries depend on the age, site, and size of the lesion [5]. The surgical techniques involve drilling either antegrade or retrograde, chondroabrasion and cancellous bone graft. Other popular methods are in use as well, including autologous chondrocyte implantation and osteochondral autograft transfer (mosaicplasty) [6] that have grown in popularity recently.

HYALOFAST® is a cell-free hyaluronic acid-based bioscaffold that stimulates new cartilage growth. The purpose of this study is to evaluate the long-term outcome of osteochondral injury of the talus when treated with a cell-free hyaluronic acid-based scaffold (Hyalofast®, Anika Therapeutics Inc., Bedford, Massachusetts, USA).

## Materials and methods

A total of seven patients with osteochondral lesions of the talus (OLT) were treated with microfracture and cell-free hyaluronic acid-based scaffold (HYALOFAST®) between 2015 and 2018 after the conservative treatment of using hyaluronic acid combined with platelet-rich plasma therapy and physiotherapy, failed. The cohort was based on the number of patients who came in for treatment at that time. Informed consent was obtained and the rights of subjects were protected. The injury was caused by trauma to the ankle due to sports injury or motor vehicle accident. All of them presented with deep ankle pain that was associated with a ligament injury. Dipaola classification (Table 1) was used to classify the lesions. This is a preoperative classification based on MRI findings. All of our patients had stage III and IV lesions.

**Table 1:** Dipaola classification used for the classification of lesions based on preoperative MRI.

Stage	Description
I	Thickening of cartilage and low signal change
II	Articular cartilage breached with low signal rim behind
III	Articular cartilage breached with high signal rim behind
IV	Loose body

**Figure 1:**
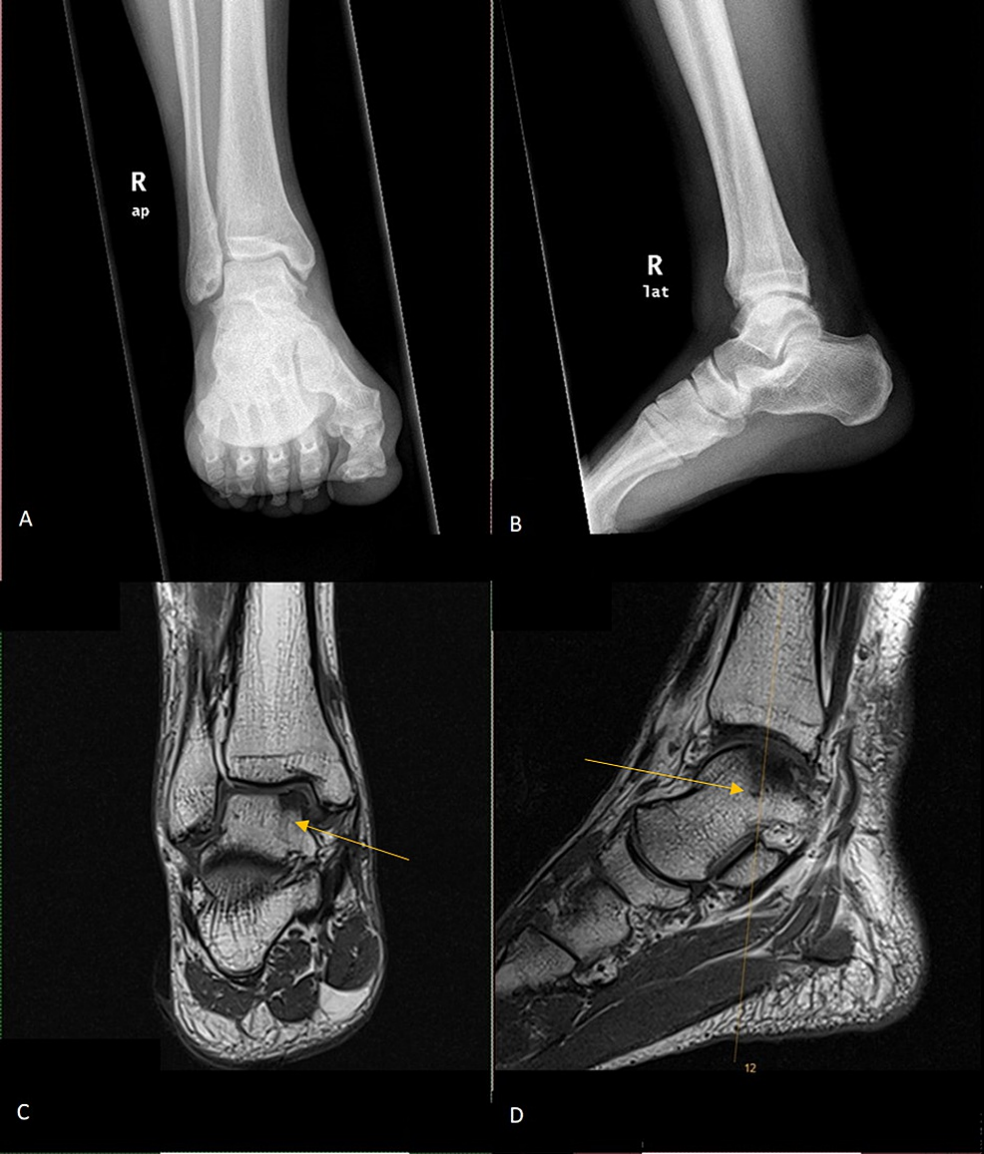
Plain radiograph of AP (A) and lateral (B) view of right ankle show fracture fragment noted in the anterior ankle joint. MRI of the right ankle (C, D) shows grade III chondromalacia (arrows) over the medial side of the talar surface with associated medial tibiotalar joint space reduction. AP: Anteroposterior

Figure 1 shows the preoperative X-ray and MRI findings, respectively, of one of the patients that underwent the procedure.

**Figure 2:**
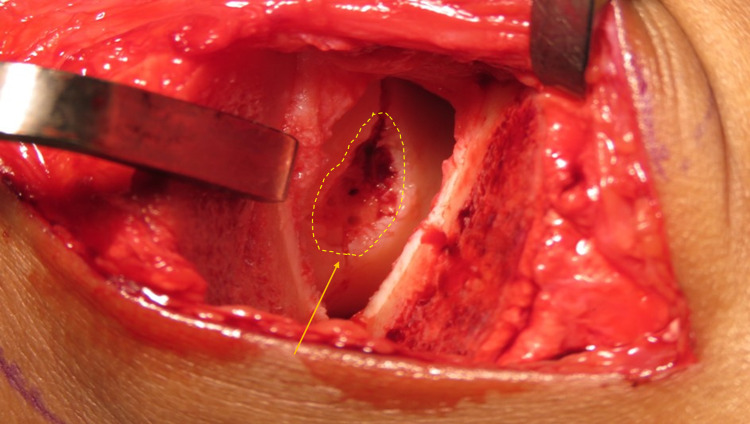
Intraoperative image shows chondral defect (arrow, dotted circle) at the medial side of talar surface with microfracture done (postmedial malleolus osteotomy).

The surgical procedure was done in the supine position under general anesthesia. The surgery was done by a single, trained foot and ankle surgeon to minimize bias. The affected lower limb was prepared and painted using povidone. Throughout the procedure, a tourniquet was applied and inflated. To visualize the chondral defect of the talus, as shown in Figure 2, an anteromedial incision was used with medial malleolar osteotomy. The area of interest was then excised and debrided. Microfracture was done and a cell-free hyaluronic acid-based scaffold (HYALOFAST®) was inserted. Tissue gel was also used to ensure the adherence of the scaffold to the base. Once hemostasis was secured, the initial medial malleolus osteotomy site was fixed with two cannulated 4.3mm screws. Medial malleolus reduction and the position of the screws were confirmed under the image intensifier.

**Figure 3:**
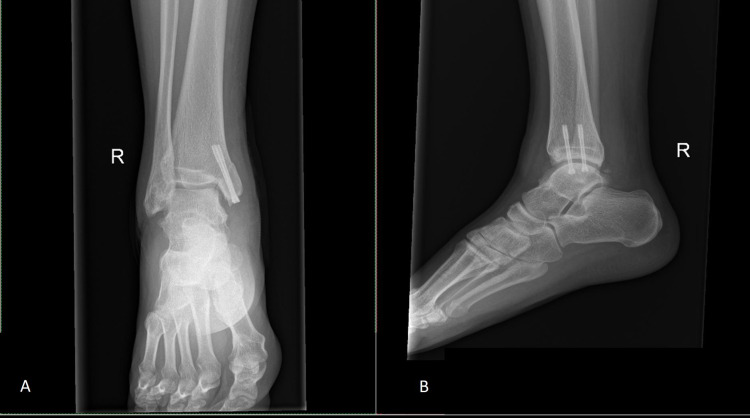
Immediate post-surgery plain radiograph of AP (A) and lateral (B) view of the right ankle show screw fixation of the medial malleolus. AP: Anteroposterior

The skin was closed by layers and the compressive dressing was done. Boot slab was applied and was kept for six weeks. Range of motion exercises of the ankle started after removal of the slab. All patients were kept non-weight bearing for eight weeks. During the follow-up, serial radiographs were taken. They were allowed to fully weight bear at 12 weeks or once there was evidence of bone healing from the plain radiograph. Patients could resume sports activities between five to six months after surgery. Figure 3 shows a postoperative X-ray of the patient that underwent this procedure.

Data were collected before surgery, and at three months, six months, 12 months, and two years postoperative. The functional assessment was done by SF36 physical functioning and mental health, and American Orthopaedic Foot and Ankle Society (AOFAS) scoring system. Range of motion (plantar flexion, dorsiflexion, inversion, and eversion) were measured. Visual analog scale (VAS) was used to measure the pain score with 0 indicating no pain and 10 indicating the worst possible pain. Data were analyzed with SPSS version 23 (IBM Corp., Armonk, NY).

## Results

This section provides a concise and precise description of the experimental results, their interpretation, as well as the experimental conclusions that can be drawn.

Table 2 shows the improvement of SF36 physical functioning from preoperative to two years postoperative. SF36 physical function showed significant improvement from preoperative when compared to one-month postoperative (p-value<0.05). Post that, SF36 physical function showed a significant improvement from six months postoperative to one year postoperative with a p-value<0.05, respectively. However, a comparison between one year postoperative and two years postoperative showed no significant value. This result shows that the treatment of cell-free hyaluronic acid-based scaffold (HYALOFAST®) can improve a patient’s physical functioning with complete recovery at the earliest by one year postoperatively.

**Table 2:** Improvement of SF36 physical functioning from preoperative to two years postoperative. SF36: Short form 36 health survey questionnaire

	SF36 Physical ( Pre-op – 1 month)	SF36 Physical (1 month – 6 months)	SF36 Physical ( 6 months – 1 year)	SF36 Physical ( 1 year – 2 years)
Mean	-15.000	-25.000	-11.429	7.857
p-value	0.051*	0.016*	0.047*	0.221

Table 3 shows the improvement of SF36 mental score from preoperative to two years postoperative. SF36 mental score shows significant improvement from preoperative till one month postoperative and up to six months postoperative with the p-value<0.05, respectively. However, there is no significant value when six months postoperative is compared to one year and two years postoperative (p-value>0.05). This result shows that a patient's mental health can be recovered completely at six months postoperatively.

**Table 3:** Improvement of SF36 mental score from preoperative to two years postoperative. SF36: Short form 36 health survey questionnaire

	SF36 Mental (Pre-op – 1 month)	SF36 Mental (1 month – 6 months)	SF36 Mental (6 months – 1 year)	SF36 Mental (1 year – 2 years)
Mean	-10.857	-16.000	-6.857	1.143
p-value	0.023*	0.024*	0.127	0.844

Table 4 shows the improvement of the AOFAS hindfoot score from preoperative to two years postoperative. There is a significant improvement of AOFAS hindfoot score from preoperative to one month postoperative, six months postoperative, and up to one year postoperative with the p-value<0.05, respectively. However, there is no significant improvement between one year postoperative and two years postoperative with a p-value>0.05. This result shows that a healthy ankle post a cell-free hyaluronic acid-based scaffold (HYALOFAST®) injection can be achieved at one year postoperatively.

**Table 4:** Improvement of AOFAS hindfoot score from preoperative up to two years postoperative. AOFAS: American Orthopaedic Foot and Ankle Society

	AOFAS Hindfoot (Pre-op – 1 month)	AOFAS Hindfoot (1 month – 6 months)	AOFAS Hindfoot (6 months – 1 year)	AOFAS Hindfoot (1 year – 2 years)
Mean	-21.714	-23.857	-10.714	-0.286
p-value	0.051*	0.032*	0.024*	0.965

Table 5 shows the improvement of the visual analog scale (VAS Score) for pain from preoperative up to two years postoperative. VAS pain score significantly reduced from preoperative to one month postoperative with p-value<0.05. Otherwise, the VAS pain scores remain insignificant from one month postoperative onwards with a p-value>0.05. It shows that the pain subsides effectively right after giving a cell-free hyaluronic acid-based scaffold (HYALOFAST®) treatment.

**Table 5:** Improvement of visual analog scale (VAS score) for pain from preoperative up to two years postoperative.

	VAS ( Pre-op – 1 month)	VAS (1 month – 6 months)	VAS ( 6 months – 1 year)	VAS ( 1 year – 2 years)
Mean	3.000	.714	.429	-.571
p-value	0.014*	0.140	0.200	0.172

## Discussion

The talus bone is the third most common region where osteochondral lesion (OCL) can develop secondary to trauma. Patients usually present after ankle injury with complaints of prolonged pain, swelling, catching, stiffness, and instability. In advanced cases, they might have a catching and grinding sensation and possibly a loose body present. This loose body can disturb joint movement and later cause joint arthrosis. If ankle pain and stiffness persist after an adequate time of conservative treatment, OLT has to be suspected. Osteochondral lesion of the talus is commonly seen in men, involving the right ankle and more on the medial side. MRI studies show medial lesions are well defined and deeper. However, lesions on the lateral side are superficial and easily displaced and, in this group, patients are symptomatic at an earlier stage.

The wide treatment strategies for OLT include nonsurgical treatment and surgical treatment. Surgical treatment includes excision of the lesion, excision, and curettage, excision combined with curettage and microfracture, placement of the cancellous bone graft, antegrade (transmalleolar) drilling, retrograde drilling, fixation, and techniques such as osteochondral autograft transfer system (OATS) and autologous chondrocyte implantation (ACI). Implant materials, fracture angles, and types of fractures are critical considerations to remember when considering an implant [7]. There is also an option of using cement and bone substitutes as complements in fracture fixation practices [8]. In our case study, we adopted medial malleolus osteotomy, microfracture, and cell-free hyaluronic acid-based scaffold (HYALOFAST®) insertion.

One of the important functions of articular cartilage is absorbing pressure across the ankle joint and because of this constant pressure, cartilage wear is expected. Pain is developed when exposed bone rubs against each other [9]. Cartilage healing potential is hampered and usually absent due to importunate friction across the joint [10]. A cell-free hyaluronic acid-based scaffold (HYALOFAST®) is a bioscaffold that stimulates new cartilage growth and is a new option in treating the OCL of the talus. This material has been vastly used in treating painful arthritic knees. A cell-free hyaluronic acid-based scaffold (HYALOFAST®) is a non-woven biodegradable hyaluronic acid (HA) based scaffold for hyaline-like cartilage regeneration. They capture mesenchymal stem cells (MSCs) to heal both chondral and OCL in the knee and ankle. The MSCs secrete paracrine factors which modulate the immune response of the host, facilitate angiogenesis, improve cell migration and survival, and prevent fibrosis [11]. Once instilled, it keeps supporting MSC attachment, proliferation, and differentiation that will plug the lesion with new cartilage. The moment cell-free hyaluronic acid-based scaffold (HYALOFAST®) breaks down; it frees more HA into the lesion, causing an embryonic-like microenvironment that further enhances the growth of the cartilage [12].

Microfracture advocates subchondral bleeding and hence fibrin clots can be formed. The unhealthy cartilage and subchondral cysts need to be removed preceding microfracture. This technique stimulates mesenchymal stem cells, growth factors, and healing proteins to the site of cartilage defect. This fibrin clot plugs in the defect and finally becomes fibrocartilage.

Treatment of OLT with cell-free hyaluronic acid-based scaffold (HYALOFAST®) in combination with microfracture showed significant improvement in VAS and AOFAS score for lesions deeper than 7mm with no postoperative complications [13]. Another study compared the outcome of arthroscopic treatment of OLT with nanofracture alone and a combination of hyaluronic acid-based scaffold and concentration of autologous bone marrow aspirate (CBMA) [14]. The cartilage quality, clinical and radiological outcomes were better in the patient treated with hyaluronic acid-based scaffold in combination with CBMA [14]. However, cell-free hyaluronic acid-based scaffold (HYALOFAST®) has a few limitations, such as failure of the scaffold to adhere to the base and cost as it is an expensive treatment that most patients cannot afford. Also, the cartilage tissue quality produced is not as strong as normal cartilage produced in the body.

The limitation of this study is the small sample size due to the lack of patients involved as most patients opt for conservative treatment. There was also a lack of validity as the results were validated by the surgeon himself. In this study, medial malleolus osteotomy, and cell-free hyaluronic acid-based scaffold (HYALOFAST®) grafting together with microfractures bring about significant improvement in pain relief, pursuing activities of daily living, sports, quality of life, and mental status in all of our patients. Plain radiograph shows new bone formation at the previous defect areas for all the patients. The medial osteotomy site for all the patients also united without any complications in regards to the surgical site or bone union. The improvement of all symptoms persists even after two years of follow-up.

## Conclusions

Medial malleolus osteotomy, cell-free hyaluronic acid-based scaffold (HYALOFAST®) grafting, and microfracture are considered relatively easy techniques. This will be a good choice for patients with sizeable cartilage deficiency. This can also provide good outcomes with low morbidity.
